# First description of adenosine production by *Gnomoniopsis smithogilvyi*, causal agent of chestnut brown rot

**DOI:** 10.1007/s11274-024-03958-4

**Published:** 2024-03-28

**Authors:** Jesús M. González-Jartín, Olga Aguín, Inés Rodríguez-Cañás, Rebeca Alvariño, María J. Sainz, Mercedes R. Vieytes, Cristina Rial, Pilar Piñón, Carmen Salinero, Amparo Alfonso, Luis M. Botana

**Affiliations:** 1 Departamento de Farmacología, Facultad de Farmacia, IDIS, Universidade de Santiago de Compostela, 15782 Santiago de Compostela, Spain; 2 Estación Fitopatolóxica Areeiro, Deputación de Pontevedra, 36153 Pontevedra, Spain; 3 Departamento de Farmacología, Facultad de Veterinaria, IDIS, Universidade de Santiago de Compostela, 27002 Lugo, Spain; 4 Departamento de Fisiología, Facultad de Veterinaria, IDIS, Universidade de Santiago de Compostela, 27002 Lugo, Spain; 5 Departamento de Producción Vegetal y Proyectos de Ingeniería, Facultad de Veterinaria, Universidade de Santiago de Compostela, 27002 Lugo, Spain

**Keywords:** *Gnomoniopsis castaneae*, Mass spectrometry, Oxasetin, Phytosphingosine, UHPLC-MS-IT-TOF

## Abstract

*Gnomoniopsis smithogilvyi* (*Gnomoniaceae*, *Diaporthales*) is the main causal agent of chestnut brown rot on sweet chestnut worldwide. The rotting of nuts leads to alterations in the organoleptic qualities and decreased fruit production, resulting in significant economic losses. In 2021, there was an important outbreak of chestnut rot in southern Galicia (Spanish northwest). The profile of secondary metabolites from *G. smithogilvyi* was studied, especially to determine its capability for producing mycotoxins, as happens with other rotting fungi, due to the possible consequences on the safety of chestnut consumption. Secondary metabolites produced by isolates of *G. smithogilvyi* growing in potato dextrose agar (PDA) medium were identified using liquid chromatography coupled with high-resolution mass spectrometry. Three metabolites with interesting pharmacological and phyto-toxicological properties were identified based on their exact mass and fragmentation patterns, namely adenosine, oxasetin, and phytosphingosine. The capacity of *G. smithogilvyi* to produce adenosine in PDA cultures was assessed, finding concentrations ranging from 176 to 834 µg/kg. Similarly, the production of mycotoxins was ruled out, indicating that the consumption of chestnuts with necrotic lesions does not pose a health risk to the consumer in terms of mycotoxins.

**Supplementary Information:**

The online version contains supplementary material available at 10.1007/s11274-024-03958-4.

## Introduction

Natural products are small molecules, primary and secondary metabolites, produced by living organisms. Plants, animals, or microorganisms serve as sources of bioactive metabolites with interesting pharmacological properties. These compounds are regularly used in the pharmaceutical and biotechnology industries, as many drugs are either based on naturally occurring molecules or are derivatives thereof (Mathur and Hoskins 2017).

Fungi constitute a large group of eukaryotic organisms with a global distribution, found in nearly all ecosystems. Metabolites derived from fungi represent a significant source of pharmaceutical compounds, such as penicillin, vancomycin, or lovastatin, some of the industrially important secondary metabolites (Devi et al. 2020). The ecological functions of many of these metabolites remain unknown; however, some can play an essential role against insect predators or competitors. Although more than 1500 compounds have been isolated from fungi, many species have not yet been studied, and, every year, new species of fungi are discovered (Keller 2019).

In 2012, an undescribed ascomycete was first reported independently by Shuttleworth et al. in Australia and Visentin et al. in Italy, albeit under different names, namely *Gnomoniopsis smithogilvyi* L.A. Shuttleworth, E.C.Y. Liew & D.I. Guest and *Gnomoniopsis castaneae* Tamietti, respectively (Shuttlewoth et al. 2012; Visentin et al. 2012). A comparative morphological and five-marker phylogenetic analysis revealed that both names represent the same species, *G. smithogilvyi* (*Gnomoniaceae*, *Diaporthales*) being the current name of the fungus, and *G. castaneae* its synonym (Shuttleworth and Guest 2017; Shuttleworth et al. 2016). This fungus was isolated as the causal agent of an unknown nut rot that began to affect up to 80% of sweet chestnut (*Castanea sativa* Mill.) production, depending on location and year, in Europe and Australia (Morales-Rodriguez et al. 2022). In 2018, *Gnomoniopsis smithogilvyi* was reported in the Portuguese region of Trás-os-Montes (Coelho and Gouveia 2021), which shares a border with Galicia in the Spanish northwest. However, it was not until September 2021 that the fungus was identified in sweet chestnut orchards in southern Galicia, causing an outbreak of chestnut brown rot (Aguín et al. 2023). The climate in this region is characterized by cold winters, high temperatures and extreme drought in the summer (MeteoGalicia 2010), conditions well tolerated by chestnuts (Freitas et al. 2021). Nevertheless, in 2021, the months of May, June and July (chestnut flowering period) experienced colder temperatures and higher rainfall compared to the 1981–2010 averages (MeteoGalicia 2022). Shuttleworth and Guest (2017) have linked climatological conditions, such as temperature and rainfall, to the infection of chestnut flowers by ascospores of *G. smithogilvyi*. In particular, rainfall during the flowering period could be responsible for an increase in the incidence of chestnut rot (Lione et al. 2015; Williams et al. 2007).

It is remarkable that *G. smithogilvyi* can also behave as an endophyte, living asymptomatically within healthy plant tissue. It may remain latent in healthy chestnuts and only cause rotting during post-harvest if optimal temperature and water conditions are present (Vettraino et al. 2021).

Several filamentous fungi that cause spoilage and/or live endophytically in food commodities, either pre-harvest or post-harvest, pose a global concern due to their ability to produce mycotoxins. These fungal secondary metabolites can be toxic to humans, other mammals, poultry, and fish mainly upon ingestion (Steyn 1995). The study of the secondary metabolites profile, particularly mycotoxins, in fungi affecting edible fruit is essential to guarantee food safety, especially when fungi live endophytically or cause small lesions that may go unnoticed, since humans or animals can consume these products. The main mycotoxigenic fungal species belong to the genera *Aspergillus*, *Penicillium* and *Fusarium*. However, it is worth noting that species within these genera are also known to produce metabolites with antifungal, anticancer, or antibacterial properties, which are of interest in therapeutics (Devi et al. 2020).

The wide array of secondary metabolites produced by fungi coupled with the high complexity of the crude extracts makes the discovery of natural products challenging. In this context, mass spectrometry (MS) technologies have proven to be a useful tool for characterizing the diversity of natural products (Louie et al. 2020). There are several types of mass analyzers, each with unique features and benefits. The ion trap (IT) serves as both a mass spectrometer, offering a wide mass range and variable mass resolution, and an ion storage device that can confine gaseous ions for a period of time, allowing subsequent fragmentation. In contrast, a time-of-flight (TOF) analyzer provides accurate mass measurement, making it useful for determining empirical formulae. However, it does not provide any information on the sequence of fragmentations (Li et al. 2007). Hybrid systems, such as high-resolution MS–IT–TOF, leverage the distinct properties of each analyzer to provide more complete information on the metabolites present in an extract (González-Jartín et al. 2017). In this context, the aim of this work was to decipher the unknown potential of *G. smithogilvyi* to produce secondary metabolites using the ultra-high-performance liquid chromatography coupled to mass spectrometry—ion trap—time of flight (UHPLC-MS-IT-TOF), a technique that allows the detection of both known and unknown metabolites, based on the determination of their exact mass.

## Material and methods

### Isolation and identification of *G. smithogilvyi* from sweet chestnut trees

In September 2021, 100 chestnuts with visible symptoms of chestnut brown rot were collected from eight orchards severely affected by the disease in southern Galicia (10–15 chestnuts per orchard). The collected samples were individually placed in paper bags and transported in portable coolers to the laboratory. In all chestnuts, each burr contained 2–3 nuts. Affected nuts, when dissected, showed brown lesions extending over most of the kernel; some nuts were mummified (Fig. S1). Small pieces of infected burrs, kernels and mummified nuts were surface disinfected using a 2% sodium hypochlorite solution for 5 min, followed by rinsing in distilled water and blotting on dry sterile filter paper. Using a sterile scalpel, pieces were plated onto potato dextrose agar (PDA) media (Sharlau, Barcelona, Spain) and incubated at 25 °C in the dark for seven days. Subsequently, mycelium growing from pieces was transferred to new PDA plates to obtain pure cultures.

The identification of isolates involved an initial reliance on morphological characteristics, followed by subsequent verification through molecular methods and phylogenetic analysis (Aguín et al. 2023).

Genomic DNA was extracted directly from visible mycelium in samples of burrs and nuts, as well as from mycelium of pure cultures grown in PDA. For molecular identification, the rDNA internal transcribed spacer (ITS) and the β-tubulin (*TUB2*) and the translation elongation factor-1α (*TEF-1* α) genes were amplified and sequenced. The primers used were ITS1F (Gardes and Bruns 1993), ITS4 (White et al. 1990), T1/Bt2b (O’Donnell et al. 1998), and EF1-728F/EF1-986R (Carbone and Kohn 1999), respectively. Subsequently, multiple sequence alignment-based phylogenetic analysis was carried out using ITS, *TUB2* and *TEF-1*α sequences.

All isolates were identified as *G. smithogilvyi*, five being selected for studying metabolite production.

### Characterization of the *G. smithogilvyi* metabolites profile

In order to establish the profile of metabolites produced by *G. smithogilvyi,* the selected isolates were subcultured in PDA at 25 °C in the dark. Control plates (PDA without fungi) were also prepared. After one week of incubation, three agar plugs (6-mm diameter) were cut from each resultant monosporic culture, and from the controls. Next, 0.5 mL of an acetonitrile/water/acetic acid mixture [49:50:1 (v/v/v)] were added to the agar plugs and the mixture was stirred in a vortex mixer for 3 min. The resulting extract was then filtered using 0.22 μm centrifugal filters (Millipore Ultrafree-MC, Billerica, MA) and frozen at − 20 °C until analysis.

The analysis of these extracts was performed using a UHPLC-MS-IT-TOF instrument from Shimadzu (Kyoto, Japan). The UHPLC system consisted of two pumps (LC-30AD), a degasser (DGU-20A), an autoinjector (SIL-10AC) with a refrigerated rack, and a column oven (CTO-10AS). Compounds were separated in a 100 mm × 2.1 mm (inside diameter), 1.8 μm, Waters ACQUITY HSS T3 column (Waters, Milford, MA) at 40 °C. Mobile phases were (A) water containing 0.1% formic acid and 5 mM ammonium formate and (B) methanol. The flow rate of the mobile phase was maintained at 0.3 mL/min, and the elution gradient (14 min) was as follows: Initially, eluent B was held at 0% for 0.5 min, and then it was increased to 50% B within 0.5 min. After 2.5 min at 50% B, the gradient was further increased to 100% B within 3 min and maintained for 3.5 min. Finally, the gradient was changed to 0% B over 0.5 min, and the column was re-equilibrated for 3.5 min. The injection volume was set at 5 μL.

The mass spectrometer, an IT-TOF instrument from Shimadzu (Kyoto, Japan), was equipped with an electrospray ionization (ESI) interface. Operating conditions were as follows: detector voltage, 1.65 kV; curved desolvation line and heat block temperature, 200 °C; nebulizing gas flow, 1.5 L/min; drying gas pressure, 105 kPa. The pressure in the ion trap was 1.8 × 10^−2^ Pa while the pressure in TOF region was 1.4 × 10^−4^ Pa. The MS method was operated in positive and negative full scan MS mode with a mass range 150–1000 Da. The ion accumulation time was set to 20 ms, with an event time of 300 ms with three repetitions. For collision-induced dissociation (CID) experiments, ions were isolated in MS^1^ with a tolerance range of 1 Dalton (Da), using argon as the collision gas with a collision energy parameter set at 50%, and the frequency parameter at 45 kHz. The mass ranges of the method in MS^2^ were adjusted for each metabolite, and the ion accumulation time was set at 30 ms.

First, the performance of the method was checked using mycotoxin standards (Fig. S2). Once the usefulness of the method for the analysis of these metabolites was confirmed, extracts were analyzed in scan mode for screening purposes. Next, the detected peaks were fragmented to study their fragmentation pathway and predict their molecular formula. These were cross-referenced in different databases in order to establish if they matched known mycotoxin or other fungal metabolites.

### Quantification of adenosine production

To quantify adenosine production by the fungi, a linear calibration curve was constructed in the extraction solvent across six concentration levels: 31.25, 62.5, 125, 250, 500, and 1000 ng/mL. To assess the matrix effect, the sample with the lowest adenosine signal was utilized to create a calibration curve in the extract, conducted in triplicate at concentrations of 125, 250, 500, 1000 and 2000 ng/mL. Simultaneously, equivalent curves were generated in solvent, and their slopes were employed to calculate the signal suppression/enhancement (SSE) caused by matrix according to the following equation:

$$SSE \left(\%\right)= \frac{Slope\,\, of\,\, spiked \,\,extracts \,\,curve}{Slope \,\,of \,\,standards \,\,curve\,\, in\,\, solvent } \times 100.$$

## Results

### Isolation and identification of *G. smithogilvyi* from sweet chestnut trees

**Fig. 1:**
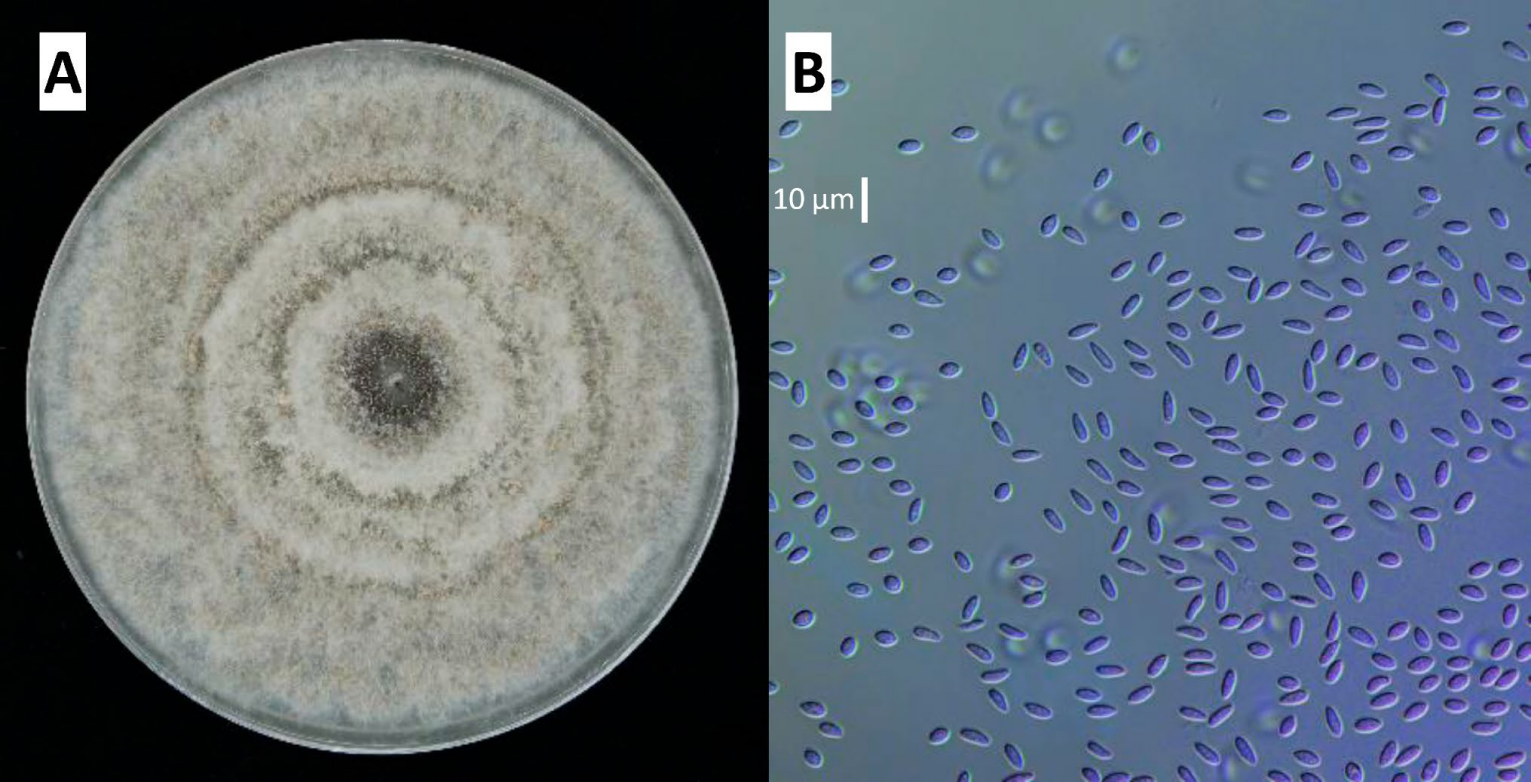
**A** Isolate of *G. smithogilvyi* on PDA. **B** Hyaline conidia of *G. smithogilvyi* (scale bar: 10 µm)

In the resulting fungal cultures on PDA medium, colonies developed in concentric circles, exhibiting a creamy white to gray or light brown woolly mycelium, with brownish to black conidiomata being abundantly produced. The conidiomata were globose to sub-globose, containing hyaline, oval, obovoid, fusoid and multi-guttulate conidia (Fig. 1). These characteristics matched those described for *G. smithogilvyi* (Shuttleworth et al. 2012). BLAST (Basic Local Alignment Search Tool) and phylogenetic analysis using sequences available at Genbank (http:/blast.ncbi.nlm.nih.gov) allowed for the conclusive identification of all isolates as *G. smithogilvyi*, with five selected for the study of metabolite production. The ITS sequences of these five isolates are deposited in the NCBI GenBank database under the accession numbers OM417081, OM417082, OR789625, OR789626, and OM417083.

### Characterization of the *G. smithogilvyi* metabolites profile

**Fig. 2:**
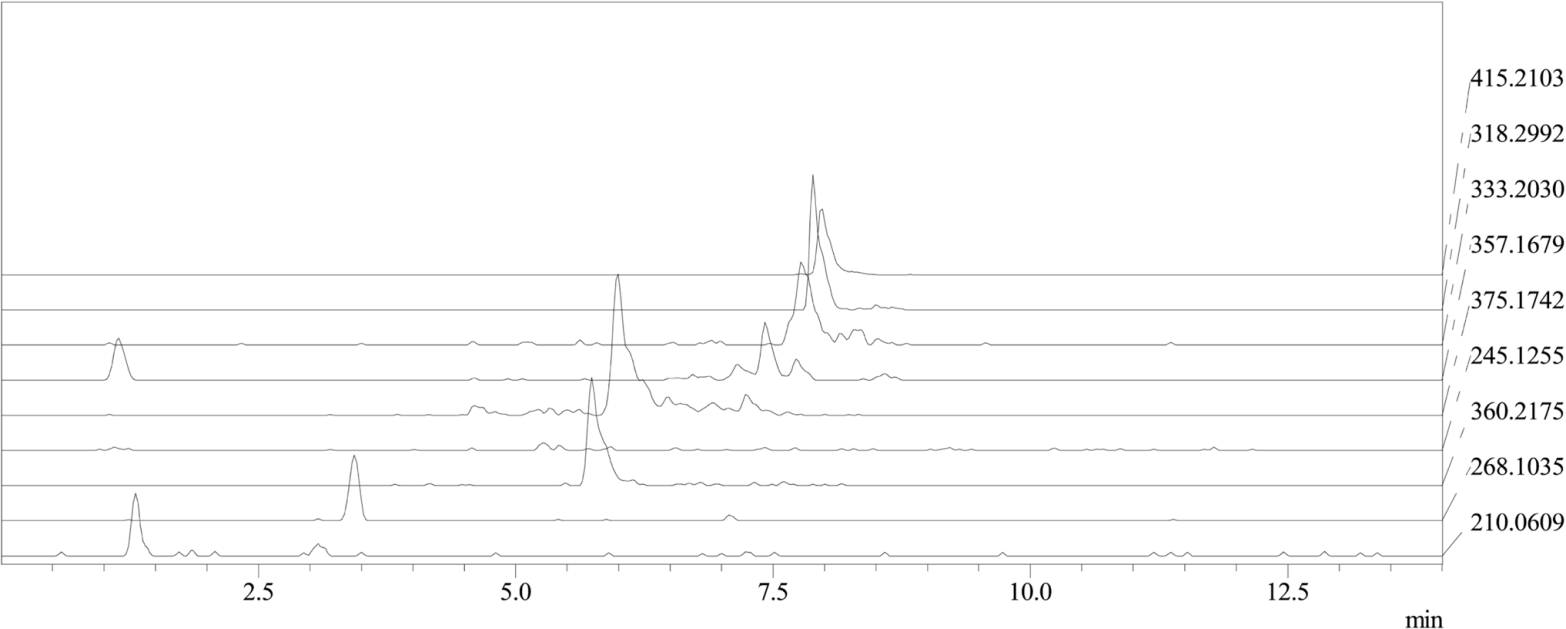
UHPLC-MS-IT-TOF chromatogram showing the main ions detected in the *G. smithogilvyi* extract

Extracts of *G. smithogilvyi* cultures were analyzed in scan mode for screening purposes. In this way, several peaks that were not present in the control sample (PDA without fungi) were identified (Fig. 2).

**Fig. 3:**
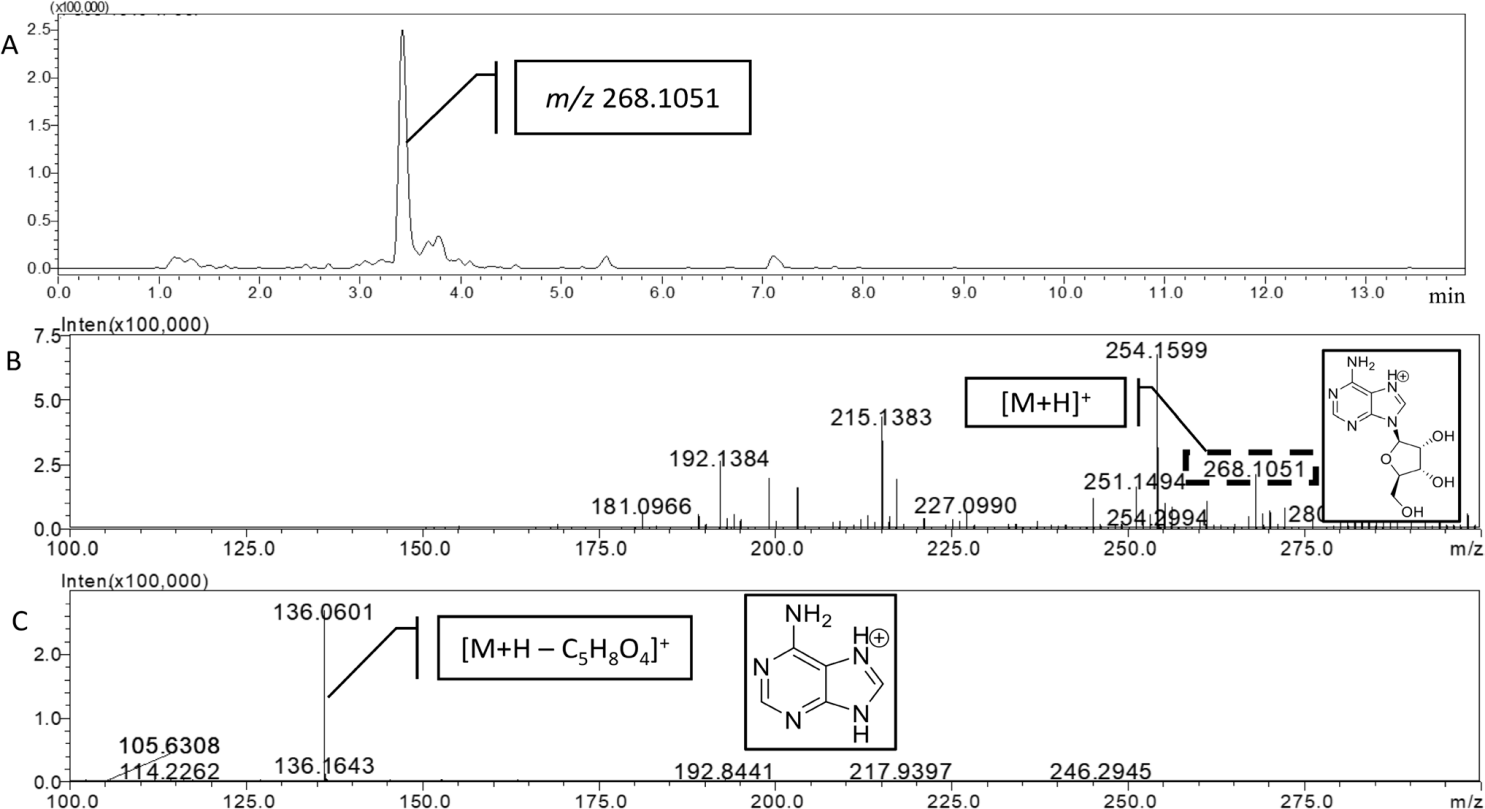
**A** UHPLC-MS-IT-TOF chromatogram of the compound eluting at 3.4 min. **B** Accurate MS^1^ spectrum of the compound. **C** Accurate MS^2^ spectrum of the ion at *m/z* 268.1051

A high intense peak was detected at 3.4 min (Fig. 3A). The extract ion chromatogram of the peak showed the ion at *m/z* 268.1051 (Fig. 3B), which was selected for CID leading to a fragment at *m/z* 136.0601 in MS^2^ spectrum (Fig. 3C). The molecular formula of the compound was predicted based on the accurate mass of parent and product ions using an ad hoc predictor software (Formula Predictor Software, Shimadzu Corp., Kyoto, Japan). In this sense, several settings were employed for an accurate prediction, including the double bond equivalents (Table S1) and a HC ratio ranging from 0.2 to 3.1. The nitrogen rule was applied, and all the isotopes (Fig. S3) were employed in the prediction. According to the obtained data, the most probable formula for the compound at *m/z* 268.1051 was C_10_H_13_N_5_O_4_, with a low mass difference (4.1 ppm) between the measured mass and the theoretical mass of the predicted formula. This formula corresponds to the [M + H]^+^ ion of adenosine, a nucleoside that is composed of adenine and d-ribose. Furthermore, the fragment ion at *m/z* 136.0601 (− 132.0450 Da) matched this molecule since it may be formed due to the loss of a glycosidic moiety [M + H-C_5_H_4_O_4_]^+^. The presence of this compound was confirmed using an analytical standard (Merck, Madrid, Spain) through the coincidence of the retention time and the fragmentation pattern between the standard and the compound present in the extract.

**Fig. 4:**
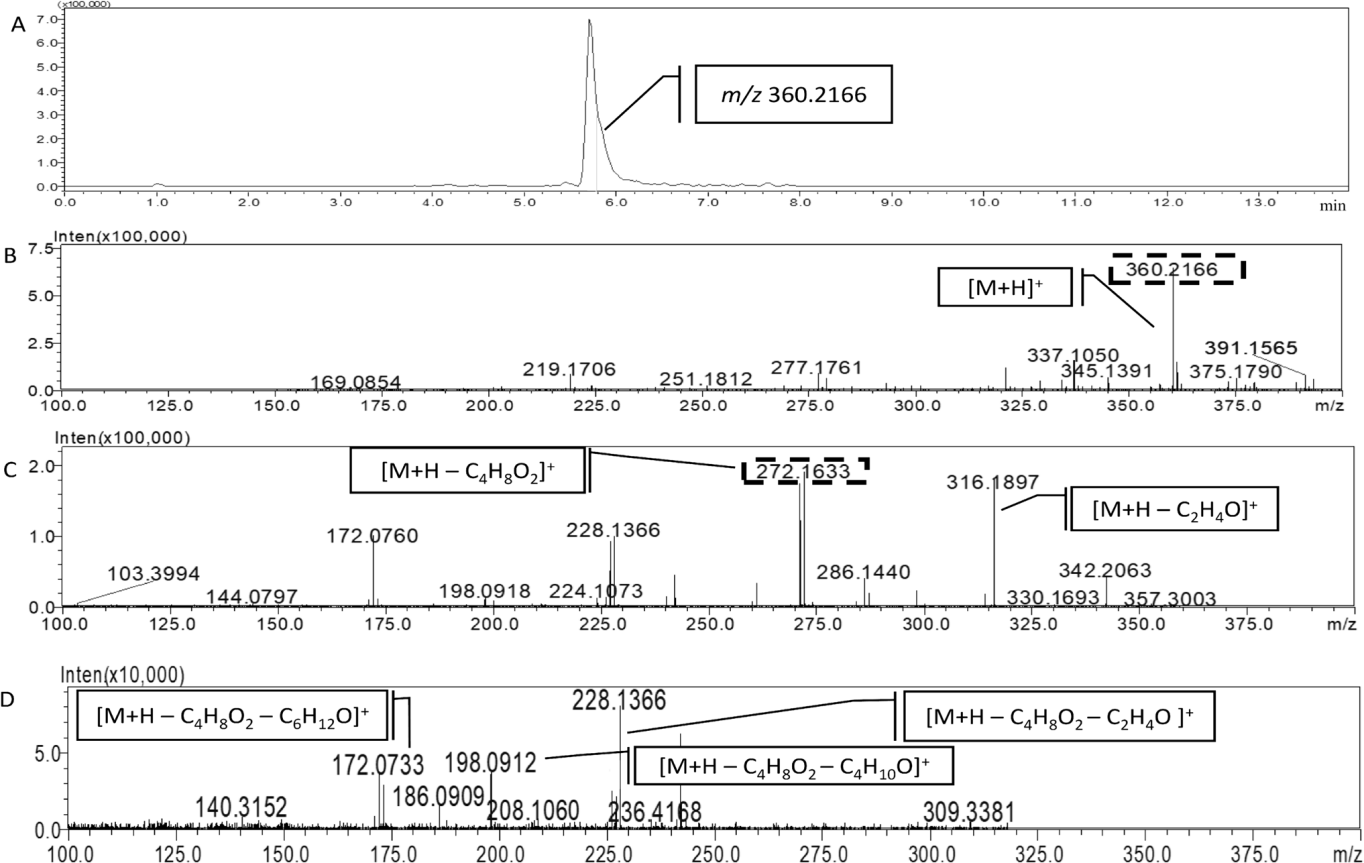
**A** UHPLC-MS-IT-TOF chromatogram of the compound eluting at 5.8 min. **B** Accurate MS^1^ spectrum of the compound. **C** Accurate MS^2^ spectrum of the ion at *m/z* 360.2166. **D** Accurate MS^3^ spectrum of the MS^2^ ion at *m/z* 272.1633

**Fig. 5:**
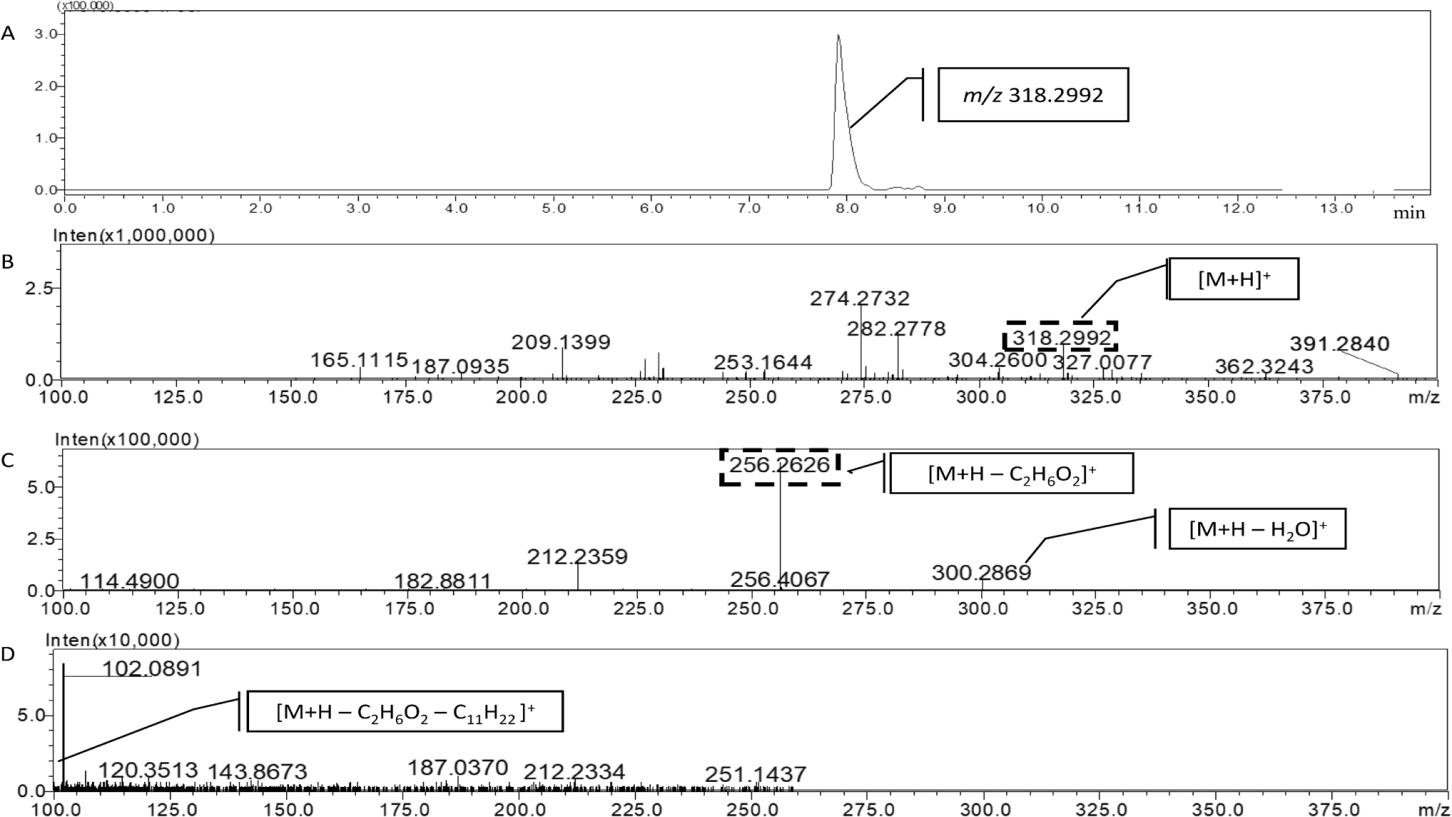
**A** UHPLC-MS-IT-TOF chromatogram of the compound eluting at 8 min. **B** Accurate MS^1^ spectrum of the compound. **C** Accurate MS^2^ spectrum of the ion at *m/z* 318.2992. **D** Accurate MS^3^ spectrum of the MS^2^ ion at *m/z* 256.2626

Next, a peak eluting at 5.8 min was studied (Fig. 4A). The extract ion chromatogram showed a high intense ion at *m/z* 360.2166 (Fig. 4B), the fragmentation of this ion led to different fragments in MS^2^ spectrum (Fig. 4C). In this sense, two main ions at *m/z* 316.1897 and *m/z* 272.1633 were observed. As shown in Fig. 4D, the subsequent fragmentation of the ion *m/z* 272.1633 led to three ions in MS^3^ (*m/z* 228.1366, *m/z* 198.0912 and *m/z* 172.0733), which were also detectable in the MS^2^ spectrum (Fig. 4C). Applying the formula predictor software, the most probable formula for the compound was determined to be C_21_H_29_NO_4_, which may correspond to oxasetin, with a low mass difference (0.83 ppm) between the theoretical and the measured masses. Subsequently, it was studied if the detected fragment ions were compatible with the structure of the proposed compound. The most probable formula for the MS^2^ ion at *m/z* 316.1897 was C_19_H_25_NO_3_ ([M + H – C_2_H_4_O]^+^) (Fig. 5C). Additionally, a high intense ion was also detected in MS^2^ at *m/z* 272.1633 ([M + H – C_4_H_8_O_2_]^+^); the subsequent fragmentation of this ion led to an MS^3^ compound at *m/*z 228.1366 [M + H – C_4_H_8_O_2_ – C_2_H_4_O]^+^, along with the ion at *m/*z 198.0912 [M + H – C_4_H_8_O_2_ –C_4_H_10_O]^+^ and at *m/*z 172.0733 [M + H – C_4_H_8_O_2_ – C_6_H_12_O]^+^ (Fig. 5D and Table S1). Consequently, the exact mass and fragmentation pattern allowed the tentative identification of the compound as oxasetin.

Finally, a third high intense compound was identified with a retention time of 8.0 min (Fig. 5A). The extract ion chromatogram displayed an ion at *m/z* 318.2992 (Fig. 5B), and its fragmentation in the MS^2^ spectrum resulted in two main fragments (Fig. 5C). Employing the exact mass of parent and products ions, the most probable formula was predicted to be C_18_H_39_NO_3_. This formula may correspond to phytosphingosine, with a low mass difference (3.46 ppm) between the theoretical and measured masses. To achieve accurate identification, the fragmentation process was examined. In the MS^2^ spectrum, the first product ion at *m/z* 300.2869 was observed, which may correspond to the loss of a water molecule ([M + H – H_2_O]^+^). Next, a high intense ion at *m/z* 256.2626 ([M + H – C_2_H_6_O_2_]^+^) was observed and its subsequent fragmentation led to a fragment in MS^3^ spectrum at *m/z* 102.0891 [M + H – C_2_H_6_O_2_ – C_11_H_22_]^+^ (Fig. 5D), which can correspond to the aminopentenol molecule present in the compound. Therefore, the exact mass of the compound and its fragmentation pattern allowed for the tentative identification as phytosphingosine.

While additional molecules were found in the extract, their identification remains elusive based on available literature. The main detected compounds and their fragments are shown in Table S2.

### Quantification of adenosine production

The quantification of adenosine production by the fungal isolates involved the use of a linear calibration curve characterized by a high regression coefficient (R) of 0.998. The data underwent correction for matrix effect, given the observed SSE factor of 55.2 ± 3.1. Following this correction, adenosine was quantified in all five isolates, with concentrations determined in triplicate as follows: 176.2 ± 28.8; 321.8 ± 16.6; 351.3 ± 40,1; 421.5 ± 177.1 and 833.8 ± 87.9 µg/kg.

## Discussion

Filamentous fungi are a promising group of microorganisms that produce bioactive metabolites with diverse chemical entities and structural functions (Nagarajan et al. 2021). *G. smithogilvyi* (syn. *G. castaneae*) has emerged as a pathogen that causes chestnut brown rot on sweet chestnut worldwide (EPPO 2022) and is currently recognized as the primary agent causing nut rot in chestnut (Lione et al. 2015). Despite its impact, the secondary metabolite profile of *G. smithogilvyi* remains unknown.. It is crucial to determine if this fungus is capable of producing mycotoxins, as observed in other rotting fungi, due to the potential consequences for the safety of chestnut consumption (Lione et al. 2019).

*Gnomoniopsis smithogilvyi* can live as an endophyte in healthy chestnuts, and if it produces toxic secondary metabolites in kernels, they could be inadvertently ingested. On the other hand, affected nuts may be rejected by the consumer when eaten raw (since brown lesions are visible); however, as the chestnut shell often shows no visible symptoms of disease, when kernels are eaten after being cooked or industrially processed (roasted in shell, into flour, etc.), necrotic lesions caused by the fungus might be also inadvertently ingested.

To elucidate the profile of mycotoxins and other secondary metabolites from *G. smithogilvyi*, five isolates were grown in PDA for a week. In this sense, a straightforward approach to study metabolite profiles of fungi is to analyze agar plugs from pure Petri dish cultures. In fact, this method is widely employed to establish the metabolites profile of fungi (González-Jartín et al. 2019b). Thus, three agar plugs were sampled from each *G. smithogilvyi* culture, and an acetonitrile/water/acetic acid mixture [49:50:1 (v/v/v)] was used as a extraction solvent, allowing the analysis of both lipophilic and hydrophilic compounds (González-Jartín et al. 2019a). TOF instruments facilitated the identification of molecules produced by the fungus based on their exact mass, an approach previously reported for the analysis of fungal metabolites (González-Jartín et al. 2017; Şenyuva et al. 2008).

As detailed below, the profile of secondary metabolites produced by *G. smithogilvyi* was studied for the first time.

The peak at *m/z* 268.1051, detected at 3.4 min (Fig. 3), was tentative identified as adenosine based on the most probable formula and the fragmentation pattern. This identification was further supported by the observation of the fragment ion at *m/z* 136.0601 in previous experiments (Jin et al. 2017). The presence of adenosine was confirmed using an analytical standard, where the retention time and fragmentation pattern of the standard matched those of the compound present in the extract. Adenosine was detected in all five isolates, with the concentrations ranging from 176.2 to 833.8 µg/kg. However, toxin production could be higher since it was not possible to correct the data with the recovery of the extraction as no blank samples were available for its calculation. In addition, this production was only determined in PDA, but it could be higher using other culture media or grown conditions (González-Jartín et al. 2019b).

Adenosine is a purine nucleoside that regulates central nervous, cardiovascular, peripheral, and immune systems. Indeed, this metabolite can be used in therapeutics due to its antiarrhythmic properties in supraventricular tachycardia (Borea et al. 2018). This compound is a major nucleoside in *Cordyceps* spp., a genus of entomopathogenic fungi in the class Ascomycetes, commonly employed in traditional Chinese medicines (Singpoonga et al. 2020; Yang et al. 2007). Adenosine has also been isolated from *Penicillium* sp., exhibiting high antioxidant activity (Yuan et al. 2014). This metabolite is the main precursor for the biosynthesis of cordycepin (Kaushik et al. 2020), the main bioactive component in *Cordyceps* (Ashraf et al. 2020); however, this metabolite was not detected in the extracts.

Next, a peak at *m/z* 360.2166 eluting at 5.8 min was studied (Fig. 4**)**. The most probable formula corresponds to oxasetin, a polyketide initially discovered from the fungus *Vaginatispora aquatica,* and later detected in *Lophiostoma* sp. (He et al. 2002; Shushni et al. 2013). Both fungi belong to the *Lophiostomaceae* family and were isolated from decaying wood submerged in a Hong Kong River and driftwood on the coast of the Baltic Sea, respectively. There are no other studies regarding the identification of this compound by mass spectrometry. However, the observed fragment ions are compatible with the structure which supports the tentatively identification of oxasetin. This compound was reported to be active in vitro against some Gram-positive bacteria, including methicillin-resistant *Staphylococcus aureus* and vancomycin-resistant *Enterococcus faecalis*, while showing no activity against *Escherichia coli* (a Gram-negative bacterium) and the yeast *Candida albicans* (He et al. 2002).

Finally, the ion at *m/z* 318.2992, with a retention time of 8.0 min, was studied (Fig. 5). The most probable formula (C_18_H_39_NO_3_) corresponds to phytosphingosine. In addition, some of the detected fragments have been reported in a previous study (Li et al. 2020). This compound is a sphingosine analogue, a structural component of sphingolipids found in fungi, plants, and some mammalian tissues (Li et al. 2006; Mota et al.). Sphingolipids are essential structural components of eukaryotic cell membranes. In filamentous fungi, glycosphingolipids are crucial for growth and pathogenesis, and, consequently, in the last few decades, they have received increasing attention for their possible role in the establishment of fungal infection (Mota et al. 2018). In particular, production of phytosphingosine seems crucial for filamentous fungal growth and viability, as has been shown for *Aspergillus nidulans* (Mota et al. 2018). On the other hand, it is well established that some sphingosine analogues inhibit sphinganine–N-acetyltransferase and ceramide synthase hampering sphingolipid biosynthesis (Möbius and Hertweck 2009). This inhibition is a common tactic employed by phytopathogenic fungi such as *Fusarium* spp., which produce fumonisins, and *Alternaria alternata,* which produces the host-specific toxin AAL-toxin (Spassieva et al. 2002; Williams et al. 2007). These phytotoxic compounds disrupt sphingolipid metabolism in susceptible tomato varieties (Abbas and Boyette 1992; Abbas and Riley 1996). Additionally, sphingolipids are important regulators in the pathogenicity of several pathogenic fungi, contributing to fungal growth in hosts under stressful conditions, maintenance of cell wall integrity, and the production of several virulence factors (Song et al. 2020). Therefore, the production of phytosphingosine by *G. smithogilvyi* may be related to its high phytotoxicity on chestnuts.

In conclusion, in this study, we first report the profile of secondary metabolites from *G. smithogilvyi*, the main causal agent of chestnut brown rot on sweet chestnut. Isolates of this pathogen were obtained from affected burrs and nuts of sweet chestnut during an outbreak of the disease in Southeastern Galicia in September 2021. Our findings demonstrate that *G. smithogilvyi* does not produce any mycotoxins. However, three secondary metabolites with interesting properties—adenosine, oxasetin and phytosphingosine—were identified based on accurate mass measurement and fragmentation patterns. The presence of adenosine was confirmed using an analytical standard. This metabolite is a well-known antiarrhythmic drug, while the production of phytosphingosine may be related to the high phytotoxicity of *G. smithogilvyi* on sweet chestnut.

### Supplementary Information

Below is the link to the electronic supplementary material.

Supplementary file1 (DOCX 2099 kb)

## Data Availability

The datasets generated during and/or analyzed during the current study are available from the corresponding author on reasonable request.
