# Supplementary material for: First description of adenosine production by Gnomoniopsis smithogilvyi, causal agent of chestnut brown rot
Source: World J Microbiol Biotechnol. 2024 Mar 28;40(5):148. doi: 10.1007/s11274-024-03958-4 (PMC10972910; doi:10.1007/s11274-024-03958-4)
Supplement: Supplementary file 1 — Supplementary file1 (DOCX 2099 kb) [file 11274_2024_3958_MOESM1_ESM.docx]

First description of adenosine production by *Gnomoniopsis smithogilvyi*, causal agent of chestnut brown rot

Jesús M. González-Jartín ^1^, Olga Aguín ^2^, Inés Rodríguez-Cañás^3^, Rebeca Alvariño ^4^, María J. Sainz ^5*^, Mercedes R. Vieytes ^4^, Cristina Rial ^2^, Pilar Piñón ^2^, Carmen Salinero ^2^, Amparo Alfonso ^3*^, Luis M. Botana ^3^

^1^Departamento de Farmacología, Facultad de Farmacia, IDIS, Universidade de Santiago de Compostela, 15782, Santiago de Compostela, Spain.

^2^ Estación Fitopatolóxica Areeiro, Deputación de Pontevedra, 36153, Pontevedra, Spain.

^3^Departamento de Farmacología, Facultad de Veterinaria, IDIS, Universidade de Santiago de Compostela, 27002, Lugo, Spain.

^4^Departamento de Fisiología, Facultad de Veterinaria, IDIS, Universidade de Santiago de Compostela, 27002, Lugo, Spain.

^5^Departamento de Producción Vegetal y Proyectos de Ingeniería, Facultad de Veterinaria, Universidade de Santiago de Compostela, 27002, Lugo, Spain.

**
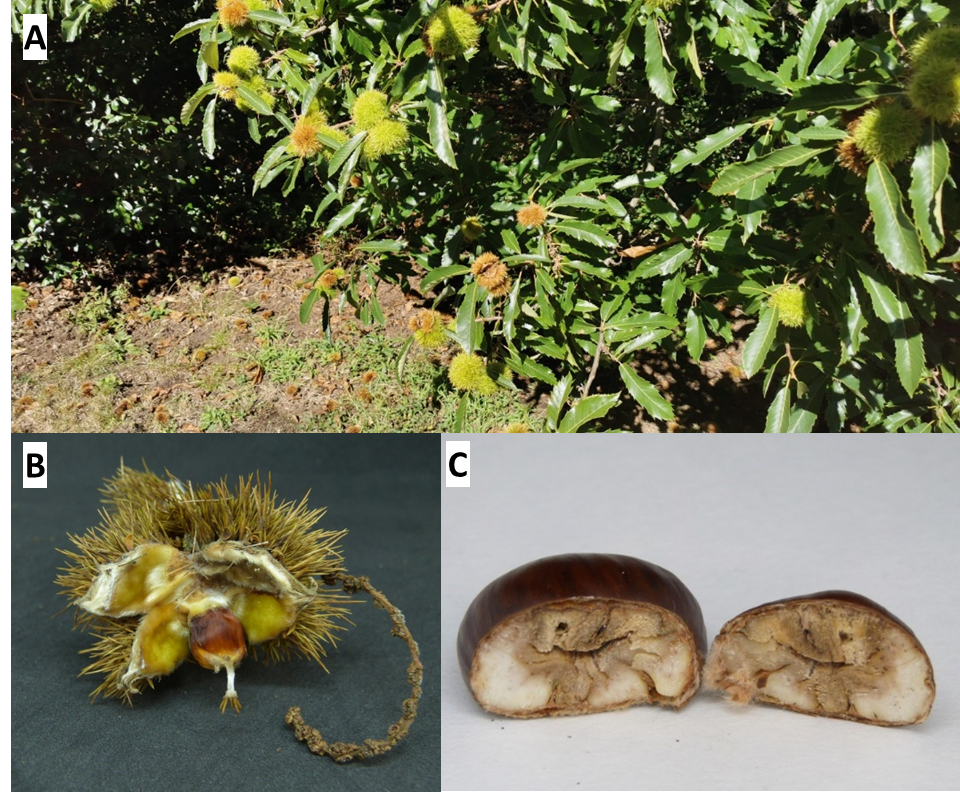
**

**Fig. S1** (A) Chestnut tree affected by chestnut brown rot; brownish immature burrs can be seen on branches and on the ground. (B) An immature burr, with a white mycelium of *Gnomoniopsis smithogilvyi* developed in its inner part, and a mummified chestnut. (C) Dissected chestnut affected by brown rot showing brown lesions extending over most of the kernel.


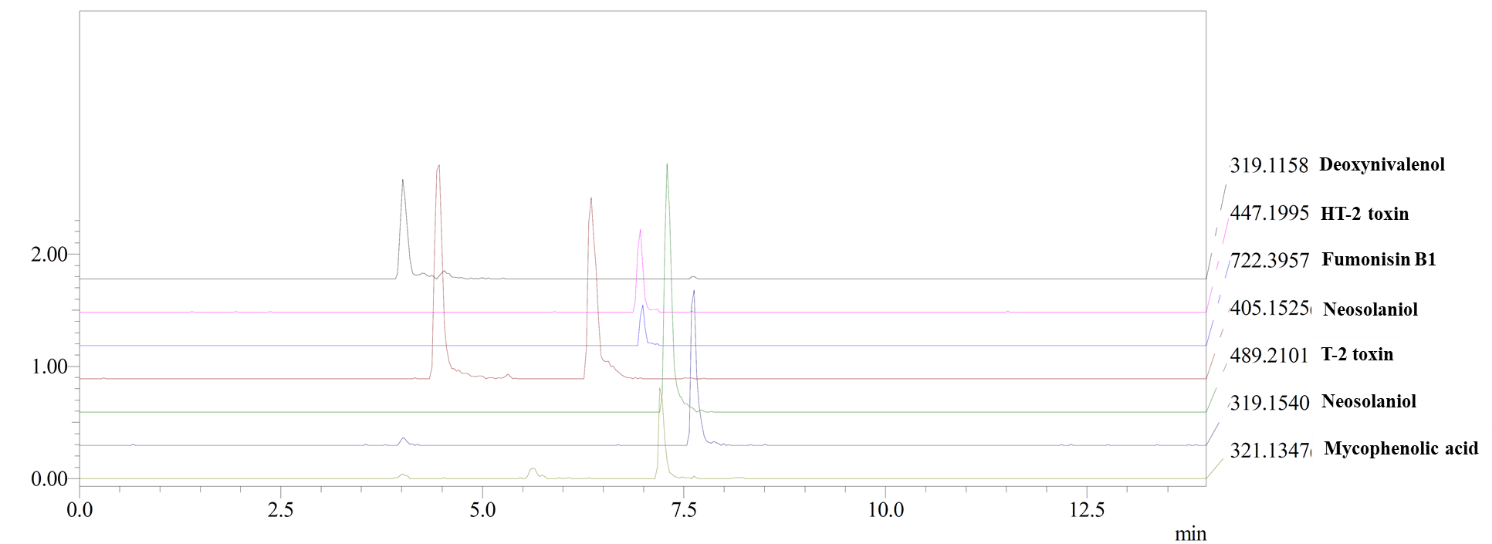


Figure S2. UHPLC-MS-IT-TOF chromatogram of a mixture of analytical standards.


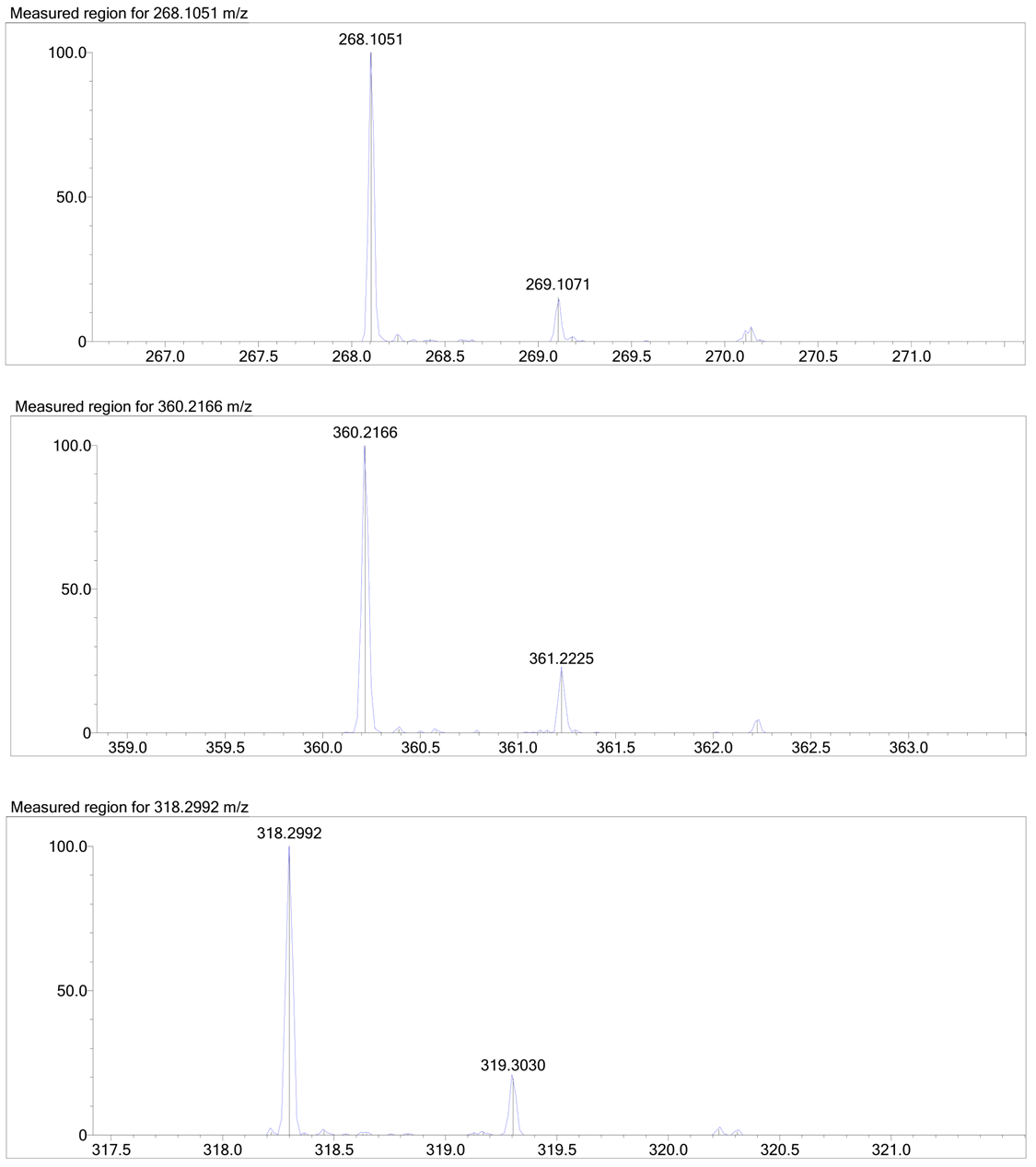


Figure S3. Isotopic pattern of the detected compounds.

| MS | Compound | Elemental composition | Experimental *m/z* | Theoretical *m/z*  (error ppm) | RDB |
| --- | --- | --- | --- | --- | --- |
| 1 | [M+H]^+^ | C_10_H_13_N_5_O_4_ | 268.1051 | 268.1040 (4.1) | 7 |
| 2 | [M+H-C_5_H_8_O_4_]^+^ | C_5_H_5_N_5_ | 136.0601 | 136.0618 (-12.4) | 6 |
|  |  |  |  |  |  |
| 1 | [M+H]^+^ | C_21_H_29_NO_4_ | 360.2166 | 360.2169 (-0.8) | 8 |
| 2 | [M+H-C_2_H_4_O]^+^ | C_19_H_25_NO_3_ | 316.1897 | 316.1907 (-3.1) | 8 |
| 2 | [M+H-C_4_H_8_O_2_]^+^ | C_17_H_21_NO_2_ | 272.1633 | 272.1645 (-4.4) | 8 |
| 3 | [M+H-C_4_H_8_O_2_- C_2_H_4_O]^+^ | C_15_H_17_NO | 228.1366 | 228.1383 (-7.4) | 8 |
| 3 | [M+H-C_4_H_8_O_2_- C_4_H_10_O]^+^ | C_13_H_11_NO | 198.0912 | 198.0913 (-0.5) | 9 |
| 3 | [M+H-C_4_H_8_O_2_- C_6_H_12_O]^+^ | C_11_H_9_NO | 172.0733 | 172.0757 (-13.9) | 8 |
|  |  |  |  |  |  |
| 1 | [M+H]^+^ | C_18_H_39_NO_3_ | 318.2992 | 318.3003 (-3.4) | 0 |
| 2 | [M+H-H_2_O]^+^ | C_18_H_38_NO_2_ | 300.2869 | 300.2897 (-9.3) | 1 |
| 2 | [M+H-C_2_H_6_O_2_]^+^ | C_16_H_33_NO | 256.2626 | 256.2635 (-3.5) | 1 |
| 3 | [M+H-C_2_H_6_O_2-_C_11_H_22_]^+^ | C_5_H_11_NO | 102.0891 | 102.0913 (-2.2) | 1 |

**Table S.1 Data for MS^n^ ions.** Precursor, elemental composition, accurately measured mass (M), theoretical *m/z* and mass errors expressed as ppm (mDa), rings and double bond equivalents (RDB)

| **[M+H]^+^ ion** | **Main fragments** |
| --- | --- |
| 421.1447 | 403.1372; 257.076 |
| 415.2103 | 295.1146; 277.1075 |
| 375.1743 | 287.1237 |
| 357.1659 | 273.1100 |
| 333.2020 | No fragments |
| 245.1255 | 120.0793 |
| 210.0609 | 193.0333; 175.0225; 147.028 |

**Table S2. Non-identified ions and their fragments detected in *Gnomoniopsis smithogilvyi* extracts**
